# Across‐country genetic evaluation of meat sheep from Ireland and the United Kingdom

**DOI:** 10.1111/jbg.12668

**Published:** 2022-02-01

**Authors:** Shauna Fitzmaurice, Joanne Conington, Kevin McDermott, Noirin McHugh, Georgios Banos

**Affiliations:** ^1^ Scotland's Rural College (SRUC) Midlothian Scotland, UK; ^2^ Teagasc, Animal & Grassland Research and Innovation Centre Moorepark Ireland; ^3^ Sheep Ireland Bandon Ireland

**Keywords:** across‐country genetic evaluations, carcass composition, lamb growth, sheep

## Abstract

Genetic evaluations in sheep have proven to be an effective way of increasing farm profitability. Much research has previously been conducted on producing within‐country genetic evaluations; however, to date, no across‐country sheep genetic evaluations have been produced between Ireland and the UK. The objective of the present study was to examine the feasibility of an across‐country genetic evaluation of live body weight and carcass composition traits for Texel sheep raised in Ireland and the UK. The benefit of genetic selection based on across‐country genetic evaluations, in comparison with within‐country genetic evaluations, was also quantified. Animal traits included early‐life and postweaning live body weights, and muscle and fat depth ultrasound measurements. Irish and UK data were combined, common animals with progeny with records in both countries were identified and a series of bivariate analyses were performed separately for each trait to produce across‐country genetic evaluations. Fixed effects included contemporary group, age at first lambing of the dam, parity of the dam (Ireland), dam age at lamb's birth (UK), a gender by age of the lamb interaction, a birth type by rearing type of the lamb interaction and country of birth of the lamb. Random effects included the animal additive genetic, dam maternal, litter common environment and residual effect. The model for postweaning weight, muscle depth and fat depth included only the animal additive genetic and litter common environmental random effects. Genetic correlations between the two countries ranged from 0.82 to 0.88 for the various traits. Across‐country breeding values were estimated for all animals and response to selection was predicted using the top 10 and top 20 sires in both within‐ and across‐country analyses for the two countries. Overall, results showed that rates of genetic gain could potentially increase from between 2.59% and 19.63% from selection based on across‐country genetic evaluations compared to within‐country evaluations alone. Across‐country evaluations are feasible and would be of significant benefit to both the Irish and UK sheep industries. In order to realize these potential gains though, there would need to be a switch in emphasis by sheep breeders towards using objective traits as their primary selection criteria.

## 1 INTRODUCTION

Pedigree sheep breeding is an international activity with high levels of trade of breeding stock occurring between countries. Growth and carcass composition traits are of high economic importance worldwide (Byrne et al., 2010; Cocks et al., 2002) and genetic selection of these traits has led to substantial economic gains in the global sheep industry (Amer et al., 2007; Conington et al., 2004; Jones et al., 2004). International genetic evaluations will allow for across‐country genetic selection of breeding stock. This will increase the rate of genetic gain achieved in comparison with within‐country selection alone due to a higher selection intensity attained from the increased number of selection candidates (Banos & Smith, 1991; Lohuis & Dekkers, 1998; Smith & Banos, 1991). To date, no across‐country genetic evaluations have been produced for sheep in the northern hemisphere. Earlier efforts have been documented in New Zealand and Australia (Young et al., 2009). On the contrary, across‐country genetic evaluations have successfully been established for both beef and dairy cattle through the development of Interbeef (Interbeef, 2020) and BREEDPLAN (Breedplan, 2021) for beef cattle and INTERBULL (Interbull, 2020) for dairy cattle. Outcomes from these initiatives already inform selective breeding programmes in multiple countries worldwide.

In sheep, large amounts of performance recording have been undertaken particularly in pedigree flocks across Ireland (Sheep Ireland) and the UK (Agriculture and Horticulture Development Board—AHDB) resulting in a high volume of data being available particularly for live body weight and carcass composition traits measured on certain common breeds. Therefore, it could be of significant advantage to pool these data together into an across‐country evaluation system to allow breeders to more accurately compare and select animals across country.

The objective of the present study was to assess the feasibility of combining lamb live body weight and carcass phenotypic records and pedigree data from Ireland and the UK in order to develop an across‐country genetic evaluation system for pedigree Texel sheep. An additional objective was to quantify the potential benefit of selection based on across‐country genetic evaluations in comparison with within‐country genetic evaluations.

## 2 MATERIALS AND METHODS

### 2.1 Data

All data used in the present study were obtained from Sheep Ireland, the Irish national database (http://www.sheep.ie) and AHDB, the UK national Sheepbreeder database (https://ahdb.org.uk/beef‐lamb). The study focussed on purebred Texel lambs. Three live body weight traits, namely preweaning weight (20–65 days of age, 12–32 kg), weaning weight (66–120 days, 20–55 kg) and postweaning weight (121–180 days, 25–75 kg) and two carcass composition traits, namely muscle depth (121–180 days, 10–44 mm) and fat depth (121–180 days, 1–23 mm) from Ireland were examined. Similar traits were examined in the UK data including early‐life body weight (40–85 days, 12–45 kg), postweaning weight (121–180 days, 25–75 kg), muscle depth (121–180 days, 10–44 mm) and fat depth (121–180 days, 0.5–8 mm). In both cases, muscle and fat depth were assessed with ultrasound measurements.

Live body weight and carcass trait data records were available on 177,307 Irish and 521,244 UK lambs born between 2010 and 2017. A number of edits were applied to the two datasets. Average daily gain was calculated from live body weight records and only lambs with a daily live weight gain of between 100 and 650 g/day were retained for analysis. Live body weight and carcass composition trait records were discarded if sire, dam, maternal grandsire or flock of birth was unknown. For live body weight and carcass composition traits in the UK, both the sire of the lamb and maternal grandsire of the lamb were required to have at least five progeny each. This was only applied to the UK data due to the high volume of data available and it allowed a more informative dataset to be created. Within the UK data, lamb records were discarded if dam age was unknown or aged >9 years. Similarly, within the Irish data dams with no known parity number or a parity number >10 were discarded. Both dam age (UK) and dam parity (Ireland) were subsequently categorized as 1, 2, 3, 4 or ≥5. Age at first lambing was defined as the age of the ewe at her first lambing. In Ireland this was whether the dam first lambed down as a ewe lamb (8–18 months of age) or a hogget (18–28 months of age) whereas in the UK data, this ranged from 1 to 3 years. Birth type was defined as the number of lambs born per lambing event; only lambs born with a birth type between 1 and 4 were retained for analysis. Rearing type was defined as the number of lambs reared per litter; only lambs with a rearing type of between 1 and 3 were retained for further analysis. Records from lambs that were artificially reared, reared by a non‐genetic dam or born as a result of embryo transfer were discarded. For all traits, lambs were allocated to a contemporary group of flock‐by‐week of weighing. Contemporary groups were only retained for further analysis if they contained five or more records. Following all data edits described 33,371 early‐life/preweaning weight records, 33,868 early‐life/weaning weight records, 25,293 postweaning weight records, 21,429 muscle depth records and 21,309 fat depth records remained across both countries (Table 1).

**TABLE 1:** Number of records (*n*), trait mean (µ) and standard deviation (*SD*), the corresponding mean age of lambs, and estimates of heritability (*h* ^2^) with standard error (SE) by country and trait, and genetic correlation of traits between countries

Trait	Country	*n*	µ (*SD*)	Age	*h* ^2^ (SE)	Genetic correlation
Preweaning weight (kg)	Ireland	11,891	20.86 (4.70)	46.59	0.19 (0.03)	0.82
Early‐life weight (kg)	UK	21,480	27.16 (6.48)	65.53	0.18 (0.03)	
Weaning weight (kg)	Ireland	12,388	36.69 (7.63)	96.92	0.30 (0.03)	0.38
Early‐life weight (kg)	UK	21,480	27.16 (6.48)	65.53	0.18 (0.03)	
Postweaning weight (kg)	Ireland	12,074	48.70 (9.47)	144.76	0.32 (0.03)	0.88
	UK	13,219	49.00 (9.24)	146.70	0.22 (0.03)	
Muscle depth (mm)	Ireland	8,810	32.59 (4.09)	146.57	0.31 (0.03)	0.85
	UK	12,619	28.69 (4.05)	146.80	0.19 (0.03)	
Fat depth (mm)	Ireland	8,782	6.10 (2.70)	146.63	0.20 (0.03)	0.85
	UK	12,527	2.45 (1.26)	146.80	0.18 (0.03)	

An international pedigree file was then produced for all animals in the original unedited dataset to allow all across‐country links to be considered in the ensuing analyses. Breeding animals that had progeny with records in both Ireland and the UK were identified to confirm the presence of genetic links between the two countries. A total of 8,392 common ancestors were found, including 1,188 common sires.

### 2.2 Genetic analysis

Combined data from Ireland and the UK were considered in a series of statistical analyses. Both carcass composition traits, namely muscle depth and fat depth, as well as postweaning weight corresponded directly across the two countries. However, whilst the early‐life body weight traits were similar in age range, they were not directly comparable between the two countries. Therefore, early‐life weight as defined in the UK had to be combined separately with preweaning and weaning weight from Ireland. The following model was used for the statistical analyses:

$$\begin{array}{c} Y=\mathrm{CG}+\mathrm{AFL}+\mathrm{Parity}+\mathrm{Dam}\;\mathrm{age}+\mathrm{Sex}*\mathrm{Age}\\ \;+\mathrm{Birth}\;\mathrm{type}*\mathrm{Rearing}\;\mathrm{type}+\mathrm{Country}+\mathrm{Animal}\\ \;+\mathrm{Dam}+\mathrm{Litter}+e \end{array}$$

Where *Y* = lamb live body weight or carcass composition record, CG, contemporary group of flock‐by‐week of weighing; AFL, age of the lamb's dam at first lambing; Parity, parity of the lamb's dam at lambing (Irish data only); Dam age, age of the lamb's dam at lambing (UK data only); Sex*Age, the interaction between the sex of the lamb and age of the lamb at recording; Birth type*Rearing type, the interaction between the birth type and rearing type of the lamb; Country, country of birth of the lamb; Animal, random additive genetic effect of the animal (lamb) including all pedigree available; Dam, random maternal effect of the lamb's dam; Litter, random common environmental effect amongst lambs in the same litter; and e, random residual effect.

The model was first applied to Irish and UK data, separately, after removing the country effect, to derive within‐country estimates of variance components and animal breeding values of individual animals in each country. Subsequently, bivariate analyses were conducted on joint across‐country data to estimate the genetic correlations between countries and breeding values of all animals in the combined dataset. In the latter analyses, all variance component estimates across country were fixed to the previously calculated within‐country estimates to allow for a direct comparison of estimated breeding values from within‐country and across‐country evaluations. Residual covariance estimates due to dam and litter effects as well as between countries were fixed to zero as no animal had phenotypic records in both countries. Estimated breeding values (EBVs) and accuracies of EBVs were derived for all animals and were expressed on the scale of each country.

All these analyses were conducted with the ASReml software (Gilmour et al., 2009).

### 2.3 Response to selection

Predicted response to selection was calculated for each studied trait in each country, separately, using the following equation (Rendel & Robertson, 1950):

$$\Delta G=i*r*\sigma_{a}$$

Where Δ*G*, rate of genetic gain achieved per generation and trait; *i*, selection intensity; *r*, accuracy of genetic evaluation; and *σ*_α_, additive genetic standard deviation for the trait in question.

This formula was used to derive the predicted response to the selection of sires based on EBVs from both across‐country and within‐country genetic evaluations. Different scenarios considered selection of the top 10 and 20 sires in each case. Only sires with a minimum EBV accuracy of 0.65 were considered in this step.

## 3 RESULTS

Phenotypic description of the studied traits is given in Table 1. This Table portrays the individual traits as included pairwise in the across‐country analyses. Overall, trait phenotypic results were relatively similar between the two countries; however, muscle depth and particularly fat depth were lower in the UK. This may be attributed to different techniques used in the two countries when measuring fat depth using the ultrasound scanning machine.

Genetic parameters from the univariate (within‐country) and bivariate (across‐country) analyses are also summarized in Table 1. For reasons that could not be determined, the bivariate analysis of muscle depth in the two countries failed to converge. Therefore, an approximate genetic correlation was derived in this case based on the correlation between EBVs of common sires calculated within country and adjusted for EBV accuracy according to Calo et al. (1973).

Genetic correlation estimates between the two countries were stronger than 0.80 in all cases except when weaning weight from Ireland was combined with early‐life weight from the UK. In this case, the weak genetic correlation (0.38) suggests that the across‐country evaluation for these traits would not be beneficial. Therefore, no further analyses were conducted for this trait combination. However, the other trait measured in Ireland at an early growth phase, preweaning weight, was highly correlated with UK early‐life weight. In the latter case, as well as for all carcass traits the strong genetic correlation estimated between the two countries warrants possible benefits from a joint genetic evaluation. Strong genetic correlations between traits also indicate that limited re‐ranking of sires would be expected between the two countries.

### 3.1 Response to selection

In order to further examine and quantify the benefit of across‐country genetic evaluation, predicted response to sire selection within and across country was estimated for each trait separately (Tables 2, 3, 4, 5). Two different selection scenarios were considered for illustration assuming selection of the top 10 and top 20 sires in each case. These numbers are generally reflective of the current selection practice in the two countries.

**TABLE 2:** Predicted response (Δ*G*) to top sire selection based on within‐ and across‐country genetic evaluation for early‐life body weight

Selection scenario	No. of sires	Proportion selected	*i*	*R*	σ	Δ*G*	%
Within country—Ireland							
Top 10 Sires	192	5.21	2.063	0.74	1.39	2.12	95.19
Top 20 Sires	192	10.42	1.755	0.74	1.39	1.81	93.51
Within country—UK							
Top 10 Sires	194	5.15	2.063	0.76	1.78	2.79	97.41
Top 20 Sires	194	10.31	1.755	0.76	1.78	2.37	95.52
Across country—Ireland							
Top 10 Sires	276	3.62	2.197	0.73	1.39	2.23	100
Top 20 Sires	276	7.25	1.9025	0.73	1.39	1.93	100
Across country—UK							
Top 10 Sires	260	3.85	2.175	0.74	1.78	2.87	100
Top 20 Sires	260	7.69	1.887	0.74	1.78	2.49	100

Note

*i*, intensity of selection; *r*, average accuracy of selection candidate EBVs; σ, genetic standard deviation of trait; % Δ*G* achievable within‐compared to across‐country selection.

**TABLE 3:** Predicted response (Δ*G*) to top sire selection based on within‐ and across‐country genetic evaluation for postweaning weight

Selection scenario	No. of sires	Proportion selected	*i*	*r*	*σ*	Δ*G*	%
Within country—Ireland							
Top 10 Sires	369	2.71	2.309	0.76	3.46	6.06	97.35
Top 20 Sires	369	5.42	2.023	0.76	3.46	5.31	96.02
Within country—UK							
Top 10 Sires	137	7.3	1.887	0.77	2.82	4.1	85.89
Top 20 Sires	137	14.59	1.554	0.77	2.82	3.38	80.37
Across country—Ireland							
Top 10 Sires	473	2.11	2.4035	0.75	3.46	6.23	100
Top 20 Sires	473	4.23	2.135	0.75	3.46	5.53	100
Across country—UK							
Top 10 Sires	325	3.08	2.2555	0.75	2.82	4.77	100
Top 20 Sires	325	6.15	1.985	0.75	2.82	4.2	100

Note

*i*, intensity of selection; *r*, average accuracy of selection candidate EBVs; *σ*, genetic standard deviation of trait; % Δ*G* achievable within‐ compared to across‐country selection.

**TABLE 4:** Predicted response (Δ*G*) to top sire selection based on within‐ and across‐country genetic evaluation for muscle depth

Selection scenario	No. of sires	Proportion selected	*i*	*r*	*σ*	Δ*G*	%
Within country—Ireland							
Top 10 Sires	279	3.58	2.197	0.76	1.66	2.77	97.00
Top 20 Sires	279	7.17	1.918	0.76	1.66	2.42	96.07
Within country—UK							
Top 10 Sires	125	8	1.858	0.75	1.3	1.81	87.42
Top 20 Sires	125	16	1.521	0.75	1.3	1.49	82.97
Across country—Ireland							
Top 10 Sires	348	2.87	2.295	0.75	1.66	2.86	100
Top 20 Sires	348	5.75	2.023	0.75	1.66	2.52	100
Across country—UK							
Top 10 Sires	246	4.07	2.154	0.74	1.3	2.08	100
Top 20 Sires	246	8.13	1.858	0.74	1.3	1.79	100

Note

*i*, intensity of selection; *r*, average accuracy of selection candidate EBVs; *σ*, genetic standard deviation of trait; % Δ*G* achievable within‐ compared to across‐country selection.

**TABLE 5:** Predicted response (Δ*G*) to top sire selection based on within‐ and across‐country genetic evaluation for fat depth

Selection scenario	No. of sires	Proportion selected	*i*	*r*	*σ*	Δ*G*	%
Within country—Ireland							
Top 10 Sires	165	6.06	1.985	0.74	0.1	0.15	93.80
Top 20 Sires	165	12.12	1.667	0.74	0.1	0.13	93.67
Within country—UK							
Top 10 Sires	119	8.4	1.831	0.75	0.42	0.57	92.99
Top 20 Sires	119	16.81	1.489	0.75	0.42	0.47	89.51
Across country—Ireland							
Top 10 Sires	228	4.39	2.116	0.73	0.1	0.16	100
Top 20 Sires	228	8.77	1.804	0.73	0.1	0.14	100
Across country—UK							
Top 10 Sires	182	5.49	2.023	0.73	0.42	0.62	100
Top 20 Sires	182	10.99	1.709	0.73	0.42	0.52	100

Note

*i*, intensity of selection; *r*, average accuracy of selection candidate EBVs; *σ*, genetic standard deviation of trait; % Δ*G* achievable within‐ compared to across‐country selection.

Difference between response to selection based on across‐ versus within‐country evaluation would be expected to be mainly due to difference in EBV accuracy and selection intensity. The average sire EBV accuracy ranged between 0.53 and 0.70 in both within‐ and across‐country genetic analyses. Although there was no overall increase in accuracy for across‐country genetic analyses there was an increase seen for sires with a substantial number of progeny in both countries. Expectedly, selection intensity was always higher when sires were selected based on the wider pool of the across‐country evaluations in comparison with selecting from the within‐country evaluations.

After the minimum accuracy threshold of 0.65 was imposed there were between 119 to 369 sires remaining in the within‐country analysis and 182–473 sires remaining for the across‐country analysis, depending on the trait. Across‐country evaluations were of benefit to both Ireland and the UK for all traits studied with a potential increase in predicted genetic gain between 2.59% (Table 2) and 19.63% (Table 3) in comparison with using within‐country evaluations alone. The lowest predicted response to selection was for early‐life body weight in the UK and the highest was for postweaning weight in the UK. Overall, predicted response to selection using across‐country genetic evaluations was of more benefit to the UK than Ireland for carcass traits although the opposite was true for live body weight in the early growth stage.

## 4 DISCUSSION

International (across‐country) genetic evaluations have already proven their worth in both the beef and dairy cattle industries with the development of Interbeef and Interbull, respectively. However, to date no international genetic evaluations have been conducted for sheep in the northern hemisphere. Interbull provides genetic evaluations for a multitude of traits for dairy cattle including production, fertility, health and conformation traits (Mark, 2004, 2005). Interbeef provides international genetic evaluations for weaning weight and calving ease in beef (ICBF, 2020; Pabiou et al., 2014); additionally, further research has been conducted on the international evaluations for carcass traits demonstrating the benefits from across‐country genetic selection in beef cattle (Englishby, 2018). The development of international genetic evaluations for sheep will be an important factor not only in improving the rate of genetic gain for growth and carcass traits but also in facilitating across‐country trade and selection of breeding stock. Therefore, in the present study we addressed this issue using Texel sheep data from Ireland and the UK by firstly determining the connectedness between the two countries and developing an international pedigree file. International EBVs were then produced for all animals and response to selection was estimated comparing the rate of genetic gain from the use of within‐country evaluations in comparison with international (across‐country) evaluations. Results from the present study show that across‐country genetic evaluations would be of significant benefit to both Irish and UK sheep industries.

### 4.1 Connectedness

Connectedness amongst sheep populations in different countries is a key component in the feasibility of conducting international genetic evaluations. This is because bias in EBV estimation is reduced as connections between flocks and separate management units are increased (Hanocq et al., 1996; Kuehn et al., 2007, 2008). Connectedness was found to be relatively high between the Irish and UK Texel populations with 1,188 sires having progeny with records in both countries. Although the number of common sires is relatively high, the number of progeny per sire is relatively low when compared to dairy cattle, where artificial insemination (AI) is the norm in breeding programmes. Furthermore, in the case of sheep, progeny of the common sires appeared in relatively few flocks. Notably, the level of connectedness observed in the present study is only reflective of the true connectedness levels amongst flocks currently participating in performance recording schemes. In order to increase overall connectedness levels amongst populations, an increase in systematic performance recording of the entire population is required in addition to a higher uptake of data recording within pedigree flocks. Moreover, an increase in the use of AI in both pedigree and commercial breeding settings would create higher levels of connectedness amongst flocks both within and across country. Further advances could also be made through the use of genomics due to the lack of depth in current pedigrees and missing relationship information associated with incorrect parentage in several breeds (Berry et al., 2019).

### 4.2 Genetic parameters

In general heritability estimates derived in the present study were higher in Ireland than in the UK. There were substantial differences between heritability estimates for postweaning weight and muscle depth between countries with Irish heritability estimates 10–12% higher than UK for these traits, respectively. These parameters are generally consistent, though, with the scientific literature (Safari & Fogarty, 2003).

The benefit of conducting international evaluations is dependent on the magnitude of the genetic correlation between countries for a trait (Mulder et al., 2005). Selection using across‐country genetic evaluations will result in a higher rate of genetic gain than national genetic evaluations when the genetic correlation between traits across country is 0.70 or greater (Mulder et al., 2005). Genetic correlations between Ireland and the UK were strongly positive for all corresponding traits analysed with the strongest correlation seen for the postweaning weight trait at 0.88. This is indicative of the similarity between the traits and production environments in both countries. Slightly stronger genetic correlation estimates were previously reported in across‐country beef evaluations for carcass traits between Ireland and the UK, ranging from 0.95 to 0.99 (Bonifazi et al., 2020; Englishby, 2018). The stronger correlations for beef cattle may be due to the traits that were chosen for the analysis. Beef carcass grading is standardized using the EUROP grading system in Europe so this trait definition may have led to the strong correlations than seen here (Craigie et al., 2012; Jakobsen et al., 2009). This may also be why, in the present study, postweaning weight showed the strongest genetic correlation as this trait is almost identically defined in both countries. Although previous across‐country carcass trait correlations reported by Englishby (2018) were stronger than those in the present study, other studies on across‐country evaluations for weaning weight in Limousin cattle were weaker at 0.76 (Venot et al., 2007), although later studies estimated across‐country genetic evaluations for the same trait in Limousin cattle to be 0.88 (Pabiou et al., 2014), which is similar to the present study. Whilst no direct across‐country genetic comparison has previously been completed for sheep, an earlier study produced across‐country correlations for selection indices between Ireland and New Zealand, which ranged from 0.66 to 0.86 for terminal and maternal indices between countries, respectively (Santos et al., 2015).

### 4.3 Response to selection

When genetic correlations between countries are sufficiently strong, combined selection of animals across country should always be on a par if not superior to within‐country selection (Smith & Banos, 1991). This was also proven to be true for sheep in the present study, with selection using across‐country evaluations proving to be superior to within‐country selection alone for all traits in both Ireland and the UK. Expected benefit in carcass related traits (postweaning weight, and muscle and fat depth) from across‐country genetic evaluations tended to be higher for the UK than in Ireland with up to a 19.63% and 6.49% predicted increase in genetic gain achieved in the UK and Ireland, respectively. This result was also seen in previous international beef evaluations between the same countries although the extent of the benefit was greater for the latter, with predicted rates of genetic gain increasing by up to 34% (Englishby, 2018). Previous studies for dairy cattle have also reported similar predicted responses to selection from international evaluations with predicted benefits of up to 17% reported by Lohuis and Dekkers (1998).

Pooling data from different countries and combining in an international dataset gives rise to a greater number of selection candidates, thus increasing selection intensity. As accuracy levels remained relatively stable in within‐ and across‐country genetic evaluations, selection intensity was deemed to be one of the most influential factors in increasing the rate of genetic gain achieved per year.

Predicted response to selection derived in the present study demonstrates the benefits for individual traits separately. At a practical level, sires are selected based on overall selection indices rather than individual trait EBV estimates. As the gain differs according to the different traits, when all growth and carcass trait EBVs are combined it is unlikely that the expected increase in genetic gain predicted on a single trait basis would be realized, whether this is operated within‐ or across‐country selection. This is an area that should be explored in the future work.

## 5 CONCLUSION

Strong links and genetic correlations between Ireland and the UK were found that would facilitate a joint genetic evaluation for Texel sheep across the two countries. Through the combination of data and pedigree records across country the present study has demonstrated that a considerable improvement can be achieved in the rate of genetic gain through the informed selection of breeding stock regardless of the country of origin. However, the actual rate of genetic gain achieved will be highly dependent on breeders future selection decisions ‐ whether this is based on the traditional method of selection using phenotype alone or if the incorporation of genetic evaluations can become the norm.

## CONFLICT OF INTEREST

None declared.
